# Objective measurement and clinical significance of IDO1 protein in hormone receptor-positive breast cancer

**DOI:** 10.1186/s40425-017-0285-7

**Published:** 2017-10-17

**Authors:** Daniel E. Carvajal-Hausdorf, Nikita Mani, Vamsidhar Velcheti, Kurt A. Schalper, David L. Rimm

**Affiliations:** 1 Department of Pathology, Yale School of Medicine, New Haven, CT USA; 2 Anatomía Patológica, Clínica Alemana- Facultad de Medicina Universidad del Desarrollo, Vitacura, Santiago Chile; 3 Translational Immuno-oncology Laboratory, Yale Cancer Center, New Haven, CT USA; 4 Solid Tumor Oncology, Taussig Cancer Institute, Cleveland Clinic, Cleveland, OH USA

**Keywords:** IDO1, Hormone receptor-positive, Survival, Tumor-infiltrating lymphocytes, Quantitative immunofluorescence

## Abstract

**Background:**

Immunostimulatory therapies targeting immune-suppressive pathways produce durable responses in advanced solid tumors. Indoleamine 2,3-dioxygenase (IDO) is the rate-limiting oxidoreductase that catalyzes the degradation of tryptophan to kynurenine. IDO induces immune tolerance by downregulating CD8+ and effector CD4+ T cell responses. IDO1, the most active isoform, is expressed in diverse tumor types and can be targeted using small molecule inhibitors. We used an objective, in situ assay to measure IDO1 in a collection of hormone receptor-positive breast cancers (HR+ BC).

**Methods:**

IDO1 protein was measured using quantitative immunofluorescence in 362 stage I-III HR+ BC represented in tissue microarrays. IDO1 levels were determined in the tumor and stroma, and stratified using median cut-point. Associations between IDO1, clinico-pathological features and CD3+, CD8+, CD20+ and FOXP3 tumor-infiltrating lymphocytes were examined using χ^2^ and Mann-Whitney tests. Survival was studied using Kaplan-Meier estimator and a proportional hazards model. All tests were two-sided.

**Results:**

IDO1 protein was observed in 76.2% of HR+ BC. There was no association between IDO1 and major clinico-pathological characteristics. Increased IDO1 correlated with decreased CD20+ infiltration (*P* = 0.0004) but not with CD3+, CD8+ or FOXP3 levels. Elevated IDO1 expression was associated with worse 20-year overall survival (log-rank *P* = 0.02, HR = 1.39, 95% C.I.: 1.05-1.82). IDO1 scores were independently associated with outcome in multivariable analysis.

**Conclusions:**

IDO1 protein is expressed in the majority of HR+ BC and is an independent negative prognostic marker. Additionally, IDO1 expression is negatively associated with tumor B-cell infiltration. Measurement of IDO1 has the potential to identify a population that might derive benefit from IDO1 blockade.

**Electronic supplementary material:**

The online version of this article (10.1186/s40425-017-0285-7) contains supplementary material, which is available to authorized users.

## Background

Hormone-receptor positive breast cancer (BC) accounts for 60-80% of all breast malignancies [1, 2]. Despite having a better prognosis than other BC subtypes, progression to metastatic disease occurs inevitably in over 20% of patients. Following the first metastasis, the median overall survival ranges between 1.3 to 2.2 years [3]. While adjuvant chemotherapeutic regimes have achieved up to 23% mortality reduction in estrogen receptor (ER)-positive, node-positive disease [4], targeting estrogen receptor or its biosynthesis has only achieved incremental improvements in disease-free survival [5–8].

Current therapeutic strategies targeting immune inhibitory molecules such as Programmed Death 1 (PD-1) and its ligand PD-L1 have shown prominent clinical responses in diverse tumor types [9–12]. Different studies have shown that tumor PD-L1 protein expression is associated with increased benefit from PD-1 axis agents in melanoma, lung adenocarcinomas and bladder tumors. In BC, PD-L1 is expressed at higher levels in hormone-receptor negative disease, suggesting a prominent role of this pathway in tumor immune evasion [9–11]. In ongoing studies of advanced triple-negative BC, PD-1 targeting agents induce anti-tumor responses in 18.5-33% of cases [12–17]. The levels of PD-L1 and lymphocyte infiltration are lower in hormone-receptor positive BC. Consistent with this notion, the objective response rates to PD-1 blocking antibodies in metastatic BC, including hormone receptor-positive cases, is only 5.4% [18]. Taken together, these data indicate that the PD-1 axis is unlikely to be a key immune evasion pathway in HR+ BC.

Indoleamine 2, 3-dioxygenase (IDO) is the rate-limiting oxidoreductase that catalyzes the degradation of tryptophan to kynurenine, leading to the production of NAD^+^ [19]. In humans, 2 IDO isoforms have been identified: IDO1 and IDO2. Both isoenzymes are encoded by different genes and share 43% sequence homology. IDO1 is catalytically more efficient than IDO2 and accounts for most of the measurable tissue IDO activity. The capacity of IDO to induce immune tolerance was demonstrated through the disruption of placental immune privilege with the IDO inhibitor 1-methyl-D-tryptophan (1-MT, indoximod, NLG-8189) [20].

IDO is primarily detected in tumor cells [21] and in myeloid antigen presenting cells in tumor-draining lymph nodes [21–26]. IDO induces local tryptophan depletion and increases inhibitory kynurenine metabolites. Additionally, depletion of amino acid triggers a rise in the amount of uncharged transfer RNA, which in turn, activates general control nonderepressible 2 (GCN2) kinase and therefore, a decrease in the rate of translation of most proteins [21–23]. Blockade of the tryptophan sufficiency signal is also known to inhibit mammalian target of rapamacin (mTOR) and an isoform of protein kinase C [27]. The end result is effector T cell anergy and proliferation arrest, as well as regulatory T cell activation in vitro and in vivo.

Several tumors express IDO including colorectal, prostatic, ovarian, endometrial and breast carcinomas; glioblastomas and melanomas [28–32]. Elevated IDO protein both in tumor cells and in tumor-draining lymph nodes is associated with adverse clinico-pathological characteristics and worse outcome [29–32]. Increased levels of IDO expression in colorectal and endometrial carcinoma have been associated with decreased CD3+ and CD8+ infiltrates [33, 34]. Additionally, in a humanized murine model, IDO expression in mesenchymal stem cells was associated with decreased tumor infiltrating T and B-cells in melanoma [35]. Ongoing phase 1 and phase 2 clinical trials are currently investigating the safety profile and efficacy of IDO inhibitors, alone or in combination with chemotherapy or other immunotherapies, in human refractory solid tumors.

Although the expression of IDO in BC and its potential as a target have been described using qualitative methods, the data are limited on the prevalence and prognostic value in hormone-receptor positive BC. Here, we objectively measured IDO1 protein levels in hormone-receptor positive breast cancer using a validated assay and quantitative immunofluorescence (QIF). We studied the association between IDO1 levels, key clinico-pathological features, tumor-infiltrating lymphocyte (TIL) levels and survival.

## Methods

### Patient cohort and tissue microarray construction

A retrospective, hormone receptor-positive (cases with positivity for estrogen and/or progesterone receptor by CLIA-cerified immunohistochemistry), stage I-III BC cohort (*N* = 362) was extracted from two Yale University BC collections (1960-2003) and evaluated in 2-fold redundancy in tissue microarray (TMA) format. All tissues were collected from primary, untreated tumors, obtained at the time of mastectomy. Clinico-pathologic information from patients was obtained from clinical records and pathology reports. The major clinico-pathological characteristics are presented in Additional file 1: Table S1. Tissue specimens were included in the corresponding TMAs as described [36]. Briefly, representative tumor areas were selected in hematoxylin/eosin–stained preparations by a pathologist and 0.6 mm cores were obtained using a needle and arrayed in a recipient block. Sections of the resultant tissue microarray are cut and transferred to glass slides for processing and staining. At time of collection for tissue microarray construction, cases with missing predictive biomarker data were retrospectively tested using CLIA-certified immunohistochemistry for estrogen receptor, progesterone receptor and HER2, and fluorescent in situ hybridization for HER2. Tissues were collected with specific consent or waived consent under the approved Yale Human Investigation committee protocol #9505008219.

### IDO1 Antibody validation and western blot

Validation of IDO1 protein assay was performed by measuring the levels of enzyme in preparations of HEK293 cells with and without exogenous IDO1 expression, as shown in Schalper et al. [37]. Cell culture conditions have been previously reported [38]. IDO1-negative HEK293 cells transfected with a tetracycline-inducible expression vector containing the full-length human IDO1 sequence (T-REx™ System; Life Technologies, Grand Island, NY) were exposed to increasing doxycycline concentrations for 72 h. Cell lysates were prepared and untreated HEK293 cells were used as negative controls. HEK293 transfectants were kindly provided by Dr. Richard Metz (NewLink Genetics, Ames, IA). Western blots from cell lysates were performed following standard conditions and IDO1 protein was detected using the mouse monoclonal antibody clone 1F8.2 (Millipore, Billerica, MA) at 1 μg/ml. Samples were blotted using β-tubulin as loading control (1:5000 dilution, Cell Signaling Technology, Inc., Danvers, MA). Relative IDO1 levels were determined by measuring the pixel densities of immunoblot bands using ImageJ software 1.48v (National Institutes of Health, Washington, DC), and were normalized to β-tubulin.

### Quantitative immunofluorescence and multiplexed TILs detection

Formalin-fixed, paraffin embedded (FFPE) samples from doxycycline-treated and untreated HEK293 IDO1 transfectants were included in a TMA and used for antibody validation using quantitative immunofluorescence (QIF). In addition, samples from human placenta were included as positive controls for endogenous IDO1. Fresh TMA cuts were deparaffinized at 60 °C for 20 min, then incubated twice in xylene for 20 min. Antigen retrieval was performed with citrate buffer pH 6.0 at 97 °C for 20 min in a pressure-boiling container (PT Module, Lab Vision, Thermo Scientific, Waltham, MA, USA). Endogenous peroxidase activity was blocked with 2.5% hydroxyl peroxide in methanol for 30 min, followed by blocking with 0.3% bovine serum albumin in 0.1 mol/L of Tris-buffered saline for 30 min at room temperature. TMA sections were incubated overnight at 4 °C with the primary IDO1 antibody clone 1F8.2 (4 μg/ml) or 1 h at room temperature for progesterone receptor (PgR) antibody clone PGR636 (1:50, Thermo-Scientific), and a polyclonal rabbit anti-cow pancytokeratin antibody (1:100, Z0622, Dako North America, Inc., Carpinteria, CA, USA). Incubation for estrogen receptor (ER) antibody clone 1D5 (1:50, Thermo-Scientific) was performed for 1 h at room temperature, with mouse cytokeratin clone AE1/AE3 (1:100, M3515, Dako). Sections were then incubated for one hour at room temperature with Alexa 546-conjugated goat anti-rabbit secondary antibody (Molecular Probes, Eugene, OR, USA) diluted 1:100 in mouse EnVision amplification reagent (Dako), or Alexa 546-conjugated goat anti-mouse secondary antibody (Molecular Probes) in 1:100 rabbit Envision amplification reagent (Dako). Cyanine 5 (Cy5) directly conjugated to tyramide (Perkin-Elmer, Waltham, MA, USA) at 1:50 dilution was used for target antibody detection. ProLong mounting medium (ProLong Gold; Molecular Probes) with 4,6-diamidino-2-phenylindole (DAPI) was used to stain nuclei.

Validation and details of the multiplexing TIL protocol are presented in Brown et al. [39]. Briefly, fresh TMA cuts were deparaffinized and subjected to antigen retrieval using EDTA buffer (Sigma-Aldrich, St. Louis, MO, USA) pH = 8.0 and boiled for 20 min at 97 °C in a pressure-boiling container (PT module, Lab Vision). Slides were then incubated with dual endogenous peroxidase block (Dako) for 10 min at room temperature and subsequently with a blocking solution containing 0.3% bovine serum albumin in 0.05% Tween solution for 30 min. Staining for pancytokeratin, CD3 (or FOXP3), CD8, and CD20 was performed using a sequential multiplexed immunofluorescence protocol with isotype-specific primary antibodies to detect epithelial tumor cells (cytokeratin, clone M3515, 1:100, Dako), T lymphocytes (CD3 IgG, 1:100, clone E272, Novus Biologicals, CO), cytotoxic T cells (CD8 IgG1, 1:250, clone C8/144B, Dako), B lymphocytes (CD20 IgG2a, 1:150, clone L26, Dako) and T regulatory cells (FOXP3 IgG, 1:100, clone D2W8E, Cell Signaling Technology). Nuclei were highlighted using DAPI. Secondary antibodies and fluorescent reagents used were goat anti-mouse Alexa488 (1:100, eBioscience, San Diego, CA, USA), anti-rabbit Envision (Dako) with biotynilated tyramide/Streptavidine-Alexa750 conjugate (Perkin-Elmer), anti-mouse IgG1 antibody (1:100, eBioscience) with Cy3-tyramide (Perkin-Elmer), anti-mouse IgG2a antibody (1:200, Abcam, MA) with Cy5-tyramide (Perkin-Elmer). Residual horseradish peroxidase activity between incubations with secondary antibodies was eliminated by exposing the slides twice for seven minutes to a solution containing benzoic hydrazide (0.136 mg) and hydrogen peroxide (50 μl). Fresh whole-tissue section cuts from morphologically normal human tonsil were included in each staining batch as positive control and to assess the inter-experimental reproducibility.

### Fluorescent measurement and scoring

QIF was performed using the AQUA (Genoptix Inc., Carlsbad, CA) method [40, 41]. Briefly, the QIF scores for IDO1 in the tumor and stromal compartments, and TILs in the stroma were calculated by dividing the target compartment pixel intensities by the area of cytokeratin positivity or by the area of absence of cytokeratin positivity with an expanded DAPI compartment, respectively. QIF scores were normalized to the exposure time and bit depth at which the images were captured, allowing scores collected at different exposure times to be comparable. All acquired histospots were visually evaluated and cases with staining artifacts or less than 1% tumor (cytokeratin staining) were excluded from the analysis.

### Cut-point selection and statistical analysis

Median cut-point was used to stratify IDO1 and TILs protein scores in low and high statuses. Protein levels were compared using Mann-Whitney’s test and linear regressions. Patient characteristics were compared using χ^2^ test. Survival functions were compared using Kaplan-Meier estimates, and statistical significance was determined using the log-rank test. Overall survival (OS) data was available for all patients. Multivariate Cox proportional hazards models including age, tumor size, lymph node status and staging as covariates was built. Statistical analysis was carried out using GraphPad Prism v6.0 software (GraphPad Software, Inc., La Jolla, CA, USA) and JMP 11 software (SAS Institute, Cary, NC, USA). All *P* values were based on two-sided tests, and all values under.05 were considered statistically significant.

## Results

As recently shown by Schalper et al. [37], the IDO1 detection assay was validated by measuring the levels in exogenous/inducible expression systems and human control samples. The reproducibility of IDO1 measurement using QIF was high, with a linear regression coefficient (*R* ^2^) between independent experiments of 0.97 in serial tissue microarray (TMA) sections (Additional file 1: Figure S1).

**Fig. 1:**
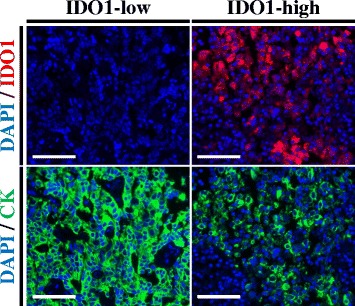
IDO1 protein measurement in hormone receptor-positive breast cancer. Fluorescent microphotographs of representative hormone receptor-positive breast cancer cases showing different levels of IDO1 protein. Staining pattern was cytoplasmic. Blue: 4,6-diamidino-2-phenylindole (DAPI). Green: Alexa-546 (Cytokeratin [CK]). Red: Cyanine-5 (IDO1). Scale bars: 100 um

**Fig. 2:**
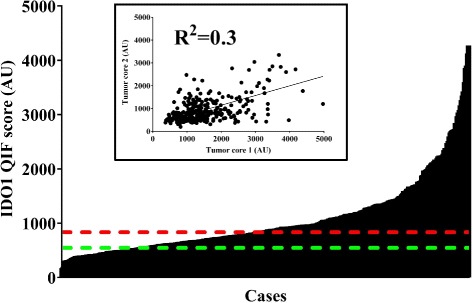
IDO1 protein levels in hormone receptor-positive cancer using automated, quantitative immunofluorescence. Bar chart shows the distribution of scores for IDO1. Red dotted line indicates the median cut-point. Green dotted line shows the visual threshold for specific IDO1 signal. Inset depicts a linear regression plot comparing 2 tumor cores in hormone-receptor positive breast cancer cases. AU: arbitrary units of fluorescence. QIF: quantitative immunofluorescence. R^2^: linear regression coefficient

Figure 1 illustrates representative cases with different IDO1 protein levels. Staining was predominantly observed in the tumor compartment, with cytoplasmic/perinuclear pattern. As shown in Fig. 2, hormone receptor-positive BCs, showed a wide range of IDO1 expression and the target was detected in 276 of 362 cases (76.2%). There was a moderate association between scores measured in different tumor areas/cores (Fig. 2 inset, *R* ^2^ = 0.3), indicating heterogeneity of expression. There was a positive correlation between IDO1 scores in the stromal compartment and tumor cells (Additional file 1: Figure S2, *R* ^2^ = 0.56). There were no associations between IDO1 levels and age, tumor size, histologic grade, nuclear grade, lymph node status or stage (Table 1). Comparison with retrospective data from our group revealed no association between ER and PgR and IDO1 expression at the protein level in HR+ BC cases (*R* ^2^ = 0.001 and *R* ^2^ = 0.004, respectively; data not shown).

**Table 1:** Major clinico-pathological variables according to IDO1 status in hormone-receptor positive breast cancer

	IDO1 status		
	IDO1-low	IDO1-high	*P* value
Age (years)			
< 50	45	47	0.81
≥ 50	136	134	
Tumor size (cm)			
≤ 2	89	93	0.36
> 2	83	71	
Histologic grade			
1-2	73	47	0.46
3	41	33	
Nuclear grade			
1-2	74	77	0.99
3	28	29	
Lymph node status			
Negative	105	117	0.17
Positive	76	63	
TNM Stage			
I-II	138	140	0.19
III	37	26	

**Fig. 3:**
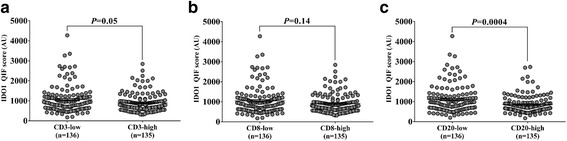
Higher IDO1 protein levels in tumor are associated with decreased B-cell infiltration in hormone receptor-positive breast cancer. Plots show levels of IDO1 protein according to CD3, CD8 and CD20 status in Yale hormone receptor-positive breast cancer cohort. TIL scores were stratified using median cut-point. The charts show mean ± standard error of the mean. Numbers under each category indicate the amount of cases in each group. AU: arbitrary unit of fluorescence. QIF: quantitative immunofluorescence

Tumor-infiltrating lymphocyte (TIL) scores for CD3 and CD8 showed the highest dynamic range in hormone receptor-positive breast cancer cases (Additional file 1: Figure S3). Additional file 1: Figure S4 shows representative microphotographs of cases with low and high levels of TILs. Elevated IDO1 was observed in tumors with low CD20 levels (*P* = 0.0004, Fig. 3c). There were no significant associations between IDO1 and CD3 or CD8 scores (Fig. 3a and b, respectively), however, there was a trend for higher IDO1 in CD3-low cases (*P* = 0.05). When IDO1 levels in the stromal compartment were stratified by TILs abundance, there were no significant associations (Additional file 1: Figure S5). Additional file 1: Figure S6A shows a representative microphotograph of T regulatory cell detection in HR+ BC by assessing FOXP3. FOXP3 levels presented a decreased dynamic range of signal, compared to other TILs makers (Additional file 1: Figure S6B). When IDO1 scores where stratified by FOXP3 status, there were no significant associations in tumor or stroma (Additional file 1: Figure S6C and D, respectively).

**Fig. 4:**
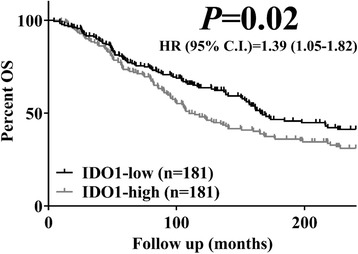
Increased IDO1 levels are associated with worse overall survival (OS) in hormone receptor positive breast cancer. Kaplan-Meier plot for 20-year OS in hormone receptor-positive breast cancer according to IDO1 status. Scores were stratified using median cut-point

In univariate Kaplan-Meier survival analysis, elevated IDO1 protein was associated with lower 20-year overall survival (log-rank *P* = 0.02, HR = 1.39, 95% C.I.: 1.05-1.82, Fig. 4). IDO1 scores were independently associated with overall survival in a multivariate Cox proportional hazards model including age, tumor size, lymph node status and stage (Table 2).

**Table 2:** Multivariate analysis of survival for IDO1 protein in the Yale hormone-receptor positive breast cancer collection

	20-year OS		
	HR	(95% C.I.)	*P* value
Age > 50 years	2.26	(1.59-3.39)	**< 0.0001**
Tumor size >2 cm	2.11	(1.59-2.81)	**< 0.0001**
Lymph node positive status	1.43	(1.00-2.01)	0.05
TNM Stage III	1.02	(0.67-1.56)	0.91
IDO1-high status	1.57	(1.19-2.09)	**0.002**

## Discussion and conclusions

Herein we report the use of a validated and reproducible assay for IDO1 protein measurement coupled to QIF in FFPE breast cancer samples. We found IDO1 protein expression in the majority of hormone receptor-positive breast tumors and negative prognostic value.

A recent article by Isla Larrain et al. [42] addressed IDO protein expression in breast cancer by qualitative chromogenic immunohistochemistry (IHC) using a commercial antibody (clone not specified). They found high IDO levels across all breast cancer subtypes, together with an association with features of aggressiveness. In contrast, a study by Soliman et al. [43], using anti-IDO antibody clone 10.1 from Millipore and semiquantitative evaluation by 2 pathologists, found higher IDO scores in ER-positive tumors. Additionally, they found that with medium/high IDO expression in ER-positive cases correlated with increased 5-year OS. This apparent discordant result is not uncommon in the pathology/biomarker literature due likely to variability in antibody performance [44] and subjective analysis of expression. None of the aforementioned reports, provided stringent assay validation data.

Estrogen signaling has been shown to increase interferon-gamma and IDO expression [45–48]. Although high IDO levels have been found in estrogen receptor positive breast cancer [42, 43], other features associated with increased interferon-gamma signaling, such as PD-L1 expression [49] and increased tumor infiltrating lymphocytes [50] have not been linked to this breast cancer subtype. We found decreased B-cell (CD20+) infiltrates and a trend for lower T-cell (CD3+) levels in cases with high IDO1 in tumor, but not in stroma. These findings are in accordance with previous studies in colorectal [33] and endometrial carcinomas [34], where IDO expression in tumor cells was negatively correlated with T-cell infiltration. We also found no difference in IDO1 protein expression between cases with low and high levels of T regulatory cell infiltration, as measured by FOXP3, similar to Soliman et al. [43]. Moreover, in a humanized murine model [35], induction of IDO expression in mesenchymal stem cells was associated with a 70% reduction in CD3+ cells in melanoma, along with lower B-cell numbers in the tumor microenvironment. In node negative breast cancer, higher levels of expression of a B-cell metagene signature were associated with improved prognosis in 3 datasets [51]. Regarding the functional state of B-cells and IDO expression, data available come from autoimmune disease models. In experimental recurrent arthritis [52], treatment with the IDO inhibitor 1-methyltryptophan (1-MT) inhibited the differentiation of autoreactive B-cells into antibody-secreting cells, but not the initial steps of activation or survival. In experimental autoimmune myasthenia gravis (EAMG), Adikari et al. [53] showed that treatment with interferon-γ-induced dendritic cells protected rats from weight loss and muscle weakness, and was associated with decreased number of plasma cells and cells expressing B-cell activating factor (BAFF). Addition of 1-MT resulted in exacerbation of EAMG symptoms. This suggests that IDO exerts tight control on B lymphocytes, especially over the population reactive to self-antigens, which might be the case of intratumoral B cells. Nevertheless, a recent report from our group in non-small cell lung cancer [37] found a significant, positive association of IDO1 protein expression with CD3+, CD8+ and CD20+ infiltration. Taken together, these data suggest a role of hormone receptor signaling in the regulation of the inflammatory milieu in the tumor microenvironment beyond interferon-gamma upregulation, possibly through prostaglandin E2, TNF-alpha, TGF-beta, IL-6 and CCL5 [37, 54]. Further studies would have to test this hypothesis.

While the prognostic role of IDO1 might be of limited clinical value, it has the potential to depict a subset of breast carcinomas that are more likely to benefit from anti-cancer immunostimulatory agents targeting IDO1. Prediction of response to therapy using tissues could be carried out at 2 levels. In the tumor microenvironment, tumor cells can constitutively express IDO or upregulate it in response to signals, such as interferon-gamma and estrogen receptor signaling, resulting decreased local anti-tumor response [21, 46, 47]. In hormone receptor-positive breast cancer, which characteristically shows lower levels of immune cell infiltration and expression of interferon gamma-induced molecules, such as PD-L1, the tumor compartment will probably have the highest levels of IDO expression. Additionally, IDO1-expressing dendritic cells might promote anergy and arrest of effector T cells and activate T regulatory cells in tumor-draining lymph nodes [24–26]. Moreover, the combined measurement of IDO and PD-L1 as well as other immune targets could support more directed immunotherapy treatments to guide clinical trials. Future studies will have to assess both tumor and tumor-draining lymph nodes for usage in companion diagnostics.

While our dataset is larger than previous studies and quantitative, it also has a number of limitations. One major limitation is that it includes only retrospectively collected tissues with different treatments and variable follow up. However, IDO1 status proved to be independent predictor of outcome, suggesting a prominent biological role. Significance is only seen in analysis of overall survival, although this may be due to the lack of data on recurrence free survival (and hence an underpowered analysis). A second issue is that the use of TMAs can underestimate or overestimate the levels of IDO1 due to tumor heterogeneity. We attempted to address this problem by testing 2 tumor cores from different regions of the tumors. We found that IDO1 is heterogeneous but shows a positive correlation between the regions sampled. Ultimately, these observations will need to be validated on conventional whole tissue sections. Lastly, our study did not include tissues from patients treated with IDO inhibitors as single-therapy or combination. First generation IDO inhibitors did not perform well as single therapy in advanced solid tumors [55, 56]. However, combination of first-in-class IDO inhibitor epacadostat (INCB024360) with anti-PD-1 pembrolizumab in stage III/IV unresectable or metastatic melanoma in Phase I trial NCT02752074 [57], demonstrated objective responses in 11/19 patients (58%), including 5 with complete response (26%) and 6 with partial response (32%). In the same patient category, the addition of indoximod to physician’s choice of checkpoint inhibitor (ipilimumab, nivolumab or pembrolizumab) in Phase II trial NLG2103 [58], showed objective responses in 31/60 patients (52%), with 6 complete responses (10%) and 25 partial responses (42%). Clinical benefit has also been recently reported in advanced renal cell [59], urothelial [60] and non-small cell lung carcinomas [61].

In summary, the measurement of IDO1 in hormone receptor-positive breast cancer has the potential to identify a population that might derive benefit from IDO1 blockade. Our study describes a method for objective and reproducible measurement IDO1 in formalin-fixed, paraffin embedded tissues with validated antibodies. We hope this work will support future efforts to test the association of tumor IDO1 expression and response to IDO targeted therapy in hormone receptor-positive breast cancer.

## Additional file

Additional file 1: Table S1. Yale hormone receptor-positive breast cancer cohort characteristics. **Figure S1.** Reproducibility of IDO1 protein measurements in serial sections using a small breast cancer TMA as control. R^2^: linear regression coefficient. **Figure S2.** Relationship between IDO1 protein levels in stroma and tumor cells. R^2^: linear regression coefficient. AU: arbitrary unit of fluorescence. **Figure S3.** Distribution of tumor-infiltrating lymphocytes in hormone receptor-positive breast cancer cases. Bar charts show the distribution of CD3 (red), CD8 (green) and CD20 (purple) quantitative immunofluorescent (QIF) signal. AU: arbitrary unit of fluorescence. QIF: quantitative immunofluorescence. **Figure S4.** Multiplexed staining for tumor-infiltrating lymphocytes in hormone receptor-positive breast cancer. Microphotographs show representative cases with low (left panel) and high (right panel) levels of stromal lymphocytic infiltration. Blue: 4,6-diamidino-2-phenylindole (DAPI). Green: fluorescein isothiocyanate (Cytokeratin [CK]). Yellow: cyanine-3 (CD8). Red: cyanine-5 (CD20). Purple: Alexa-750 (CD3). Scale bars: 100 um. **Figure S5.** IDO1 protein levels in stroma according to tumor-infiltrating lymphocyte levels. Plots show levels of IDO1 protein in the stromal compartment according to CD3, CD8 and CD20 status in Yale hormone receptor-positive breast cancer cohort. TIL scores were stratified using median cut-point. The charts show mean ± standard error of the mean. Numbers under each category indicate the amount of cases in each group. AU: arbitrary unit of fluorescence. QIF: quantitative immunofluorescence. **Figure S6.** T regulatory cells in hormone receptor-positive breast cancer, as measured by FOXP3. **A**. Representation microphotograph depicting FOXP3 staining in a breast cancer case. Blue: 4,6-diamidino-2-phenylindole (DAPI). Green: fluorescein isothiocyanate (Cytokeratin [CK]). Red: Alexa-750 (FOXP3). Scale bar: 100 um. **B**. Distribution of FOXP3 scores in 202 cases from HR+ BC cohort. Red dotted line indicates the median cut-point. **C-D**. IDO1 protein levels according to FOXP3 status in tumor (**C**) and stroma (**D**). The charts show mean ± standard error of the mean. Numbers under each category indicate the amount of cases in each group. AU: arbitrary unit of fluorescence. QIF: quantitative immunofluorescence. (DOCX 6622 kb)

## Abbreviations

DAPI: 4,6-diamidino-2-phenylindole
EDTA: Ethylenediaminetetraacetic acid
ER: Estrogen receptor
FFPE: Formalin-fixed, paraffin-embedded
GCN2: General control nonderepressible 2
HR+ BC: Hormone receptor-positive breast cancer
IDO: Indoleamine 2,3 dioxygenase
IHC: Immunohistochemistry
mTOR: Mammalian target of rapamacin
NAD: Nicotinamide adenine dinucleotide
OS: Overall survival
PD-1: Program death 1
PD-L1: Program death ligand 1
QIF: Quantitative immunofluoresce
R^2^: Linear regression coefficient
RNA: Ribonucleic acid
TIL: Tumor-infiltrating lymphocytes
TMA: Tissue microarray

## References

[1] Harvey JM, Clark GM, Osborne CK, Allred DC (1999). Estrogen receptor status by immunohistochemistry is superior to the ligand-binding assay for predicting response to adjuvant endocrine therapy in breast cancer. J Clin Oncol Off J Am Soc Clin Oncol.

[2] Mohsin SK, Weiss H, Havighurst T, Clark GM, Berardo M, Roanh le D (2004). Progesterone receptor by immunohistochemistry and clinical outcome in breast cancer: a validation study. Mod Pathol.

[3] Kennecke H, Yerushalmi R, Woods R, Cheang MC, Voduc D, Speers CH (2010). Metastatic behavior of breast cancer subtypes. J Clin Oncol.

[4] Berry DA, Cirrincione C, Henderson IC, Citron ML, Budman DR, Goldstein LJ (2006). Estrogen-receptor status and outcomes of modern chemotherapy for patients with node-positive breast cancer. JAMA.

[5] Early Breast Cancer Trialists' Collaborative Group (1998). Tamoxifen for early breast cancer: an overview of the randomised trials. Lancet.

[6] Kaufmann M, Jonat W, Hilfrich J, Eidtmann H, Gademann G, Zuna I (2007). Improved overall survival in postmenopausal women with early breast cancer after anastrozole initiated after treatment with tamoxifen compared with continued tamoxifen: the ARNO 95 study. J Clin Oncol.

[7] Pagani O, Regan MM, Walley BA, Fleming GF, Colleoni M, Lang I (2014). Adjuvant exemestane with ovarian suppression in premenopausal breast cancer. N Engl J Med.

[8] Cuzick J, Sestak I, Baum M, Buzdar A, Howell A, Dowsett M (2010). Effect of anastrozole and tamoxifen as adjuvant treatment for early-stage breast cancer: 10-year analysis of the ATAC trial. Lancet Oncol.

[9] Wimberly H, Brown JR, Schalper K, Haack H, Silver MR, Nixon C (2015). PD-L1 expression correlates with tumor-infiltrating lymphocytes and response to Neoadjuvant chemotherapy in breast cancer. Cancer Immunol Res.

[10] Schalper KA, Velcheti V, Carvajal D, Wimberly H, Brown J, Pusztai L (2014). In situ tumor PD-L1 mRNA expression is associated with increased TILs and better outcome in breast carcinomas. Clin Cancer Res.

[11] Mittendorf EA, Philips AV, Meric-Bernstam F, Qiao N, Wu Y, Harrington S (2014). PD-L1 expression in triple-negative breast cancer. Cancer Immunol Res.

[12] Topalian SL, Hodi FS, Brahmer JR, Gettinger SN, Smith DC, McDermott DF (2012). Safety, activity, and immune correlates of anti-PD-1 antibody in cancer. N Engl J Med.

[13] Brahmer JR, Tykodi SS, Chow LQ, Hwu WJ, Topalian SL, Hwu P (2012). Safety and activity of anti-PD-L1 antibody in patients with advanced cancer. N Engl J Med.

[14] Herbst RS, Soria JC, Kowanetz M, Fine GD, Hamid O, Gordon MS (2014). Predictive correlates of response to the anti-PD-L1 antibody MPDL3280A in cancer patients. Nature.

[15] Powles T, Eder JP, Fine GD, Braiteh FS, Loriot Y, Cruz C (2014). MPDL3280A (Anti-PD-L1) treatment leads to clinical activity in metastatic bladder cancer. Nature.

[16] Leisha A. Emens FSB, Philippe Cassier, Jean-Pierre Delord, Joseph Paul Eder, Marcella Fasso, Yuanyuan Xiao, Yan Wang, Luciana Molinero, Daniel S. Chen, Ian Krop. Inhibition of PD-L1 by MPDL3280A leads to clinical activity in patients with metastatic triple-negative breast cancer (TNBC). American Association for Cancer Research Annual Meeting. Philadelphia, PA. USA; 2015.

[17] Nanda R, Chow LQ, Dees EC, Berger R, Gupta S, Geva R (2016). Pembrolizumab in patients with advanced triple-negative breast cancer: phase Ib KEYNOTE-012 study. J Clin Oncol.

[18] Dirix LY Y TI, Nikolinakos P, Jerusalem G, Arkenau H-T, Hamilton EP P, von Heydebreck A, Grote H-J, Chin K and, ME L. Avelumab (MSB0010718C), an anti-PD-L1 antibody, in patients with locally advanced or metastatic breast cancer: A phase Ib JAVELIN solid tumor trial. San Antonio Breast Cancer Symposium. San Antonio, TX. USA; 2015.

[19] Macchiarulo A, Camaioni E, Nuti R, Pellicciari R (2009). Highlights at the gate of tryptophan catabolism: a review on the mechanisms of activation and regulation of indoleamine 2,3-dioxygenase (IDO), a novel target in cancer disease. Amino Acids.

[20] Munn DH, Zhou M, Attwood JT, Bondarev I, Conway SJ, Marshall B (1998). Prevention of allogeneic fetal rejection by tryptophan catabolism. Science.

[21] Munn DH, Mellor AL (2007). Indoleamine 2,3-dioxygenase and tumor-induced tolerance. J Clin Invest.

[22] Munn DH, Mellor AL (2013). Indoleamine 2,3 dioxygenase and metabolic control of immune responses. Trends Immunol.

[23] Munn DH, Sharma MD, Baban B, Harding HP, Zhang Y, Ron D (2005). GCN2 Kinase in T cells mediates proliferative arrest and anergy induction in response to indoleamine 2,3-dioxygenase. Immunity.

[24] Munn DH, Sharma MD, Hou D, Baban B, Lee JR, Antonia SJ (2004). Expression of indoleamine 2,3-dioxygenase by plasmacytoid dendritic cells in tumor-draining lymph nodes. J Clin Invest.

[25] Munn DH, Sharma MD, Lee JR, Jhaver KG, Johnson TS, Keskin DB (2002). Potential regulatory function of human dendritic cells expressing indoleamine 2,3-dioxygenase. Science.

[26] Sharma MD, Baban B, Chandler P, Hou DY, Singh N, Yagita H (2007). Plasmacytoid dendritic cells from mouse tumor-draining lymph nodes directly activate mature Tregs via indoleamine 2,3-dioxygenase. J Clin Invest.

[27] Metz R, Rust S, Duhadaway JB, Mautino MR, Munn DH, Vahanian NN (2012). IDO inhibits a tryptophan sufficiency signal that stimulates mTOR: a novel IDO effector pathway targeted by D-1-methyl-tryptophan. Oncoimmunology.

[28] Uyttenhove C, Pilotte L, Theate I, Stroobant V, Colau D, Parmentier N (2003). Evidence for a tumoral immune resistance mechanism based on tryptophan degradation by indoleamine 2,3-dioxygenase. Nat Med.

[29] Wainwright DA, Balyasnikova IV, Chang AL, Ahmed AU, Moon KS, Auffinger B (2012). IDO expression in brain tumors increases the recruitment of regulatory T cells and negatively impacts survival. Clin Cancer Res.

[30] Okamoto A, Nikaido T, Ochiai K, Takakura S, Saito M, Aoki Y (2005). Indoleamine 2,3-dioxygenase serves as a marker of poor prognosis in gene expression profiles of serous ovarian cancer cells. Clin Cancer Res.

[31] Takao M, Okamoto A, Nikaido T, Urashima M, Takakura S, Saito M (2007). Increased synthesis of indoleamine-2,3-dioxygenase protein is positively associated with impaired survival in patients with serous-type, but not with other types of, ovarian cancer. Oncol Rep.

[32] Ino K, Yoshida N, Kajiyama H, Shibata K, Yamamoto E, Kidokoro K (2006). Indoleamine 2,3-dioxygenase is a novel prognostic indicator for endometrial cancer. Br J Cancer.

[33] Brandacher G, Perathoner A, Ladurner R, Schneeberger S, Obrist P, Winkler C (2006). Prognostic value of indoleamine 2,3-dioxygenase expression in colorectal cancer: effect on tumor-infiltrating T cells. Clin Cancer Res.

[34] Ino K, Yamamoto E, Shibata K, Kajiyama H, Yoshida N, Terauchi M (2008). Inverse correlation between tumoral indoleamine 2,3-dioxygenase expression and tumor-infiltrating lymphocytes in endometrial cancer: its association with disease progression and survival. Clin Cancer Res.

[35] Ling W, Zhang J, Yuan Z, Ren G, Zhang L, Chen X (2014). Mesenchymal stem cells use IDO to regulate immunity in tumor microenvironment. Cancer Res.

[36] Giltnane JM, Rimm DL (2004). Technology insight: identification of biomarkers with tissue microarray technology. Nat Clin Pract Oncol.

[37] Schalper KA, Carvajal-Hausdorf D, McLaughlin J, Altan M, Velcheti V, Gaule P (2017). Differential expression and significance of PD-L1, IDO-1 and B7-H4 in human lung cancer. Clin Cancer Res.

[38] Metz R, Duhadaway JB, Kamasani U, Laury-Kleintop L, Muller AJ, Prendergast GC (2007). Novel tryptophan catabolic enzyme IDO2 is the preferred biochemical target of the antitumor indoleamine 2,3-dioxygenase inhibitory compound D-1-methyl-tryptophan. Cancer Res.

[39] Brown JR, Wimberly H, Lannin DR, Nixon C, Rimm DL, Bossuyt V (2014). Multiplexed quantitative analysis of CD3, CD8, and CD20 predicts response to neoadjuvant chemotherapy in breast cancer. Clin Cancer Res.

[40] Camp RL, Chung GG, Rimm DL (2002). Automated subcellular localization and quantification of protein expression in tissue microarrays. Nat Med.

[41] Neumeister VM, Anagnostou V, Siddiqui S, England AM, Zarrella ER, Vassilakopoulou M (2012). Quantitative assessment of effect of preanalytic cold ischemic time on protein expression in breast cancer tissues. J Natl Cancer Inst.

[42] Isla Larrain MT, Rabassa ME, Lacunza E, Barbera A, Creton A, Segal-Eiras A (2014). IDO is highly expressed in breast cancer and breast cancer-derived circulating microvesicles and associated to aggressive types of tumors by in silico analysis. Tumour Biol.

[43] Soliman H, Rawal B, Fulp J, Lee JH, Lopez A, Bui MM (2013). Analysis of indoleamine 2-3 dioxygenase (IDO1) expression in breast cancer tissue by immunohistochemistry. Cancer Immunol Immunother.

[44] Rimm D, Schalper K, Pusztai L (2014). Unvalidated antibodies and misleading results. Breast Cancer Res Treat.

[45] Fox HS, Bond BL, Parslow TG (1991). Estrogen regulates the IFN-gamma promoter. J Immunol.

[46] Karpuzoglu-Sahin E, Zhi-Jun Y, Lengi A, Sriranganathan N, Ansar AS (2001). Effects of long-term estrogen treatment on IFN-gamma, IL-2 and IL-4 gene expression and protein synthesis in spleen and thymus of normal C57BL/6 mice. Cytokine.

[47] Karpuzoglu-Sahin E, Hissong BD, Ansar AS (2001). Interferon-gamma levels are upregulated by 17-beta-estradiol and diethylstilbestrol. J Reprod Immunol.

[48] Xiao BG, Liu X, Link H (2004). Antigen-specific T cell functions are suppressed over the estrogen-dendritic cell-indoleamine 2,3-dioxygenase axis. Steroids.

[49] Baptista MZ, Sarian LO, Derchain SF, Pinto GA, Vassallo J (2016). Prognostic significance of PD-L1 and PD-L2 in breast cancer. Hum Pathol.

[50] Stanton SE, Adams S, Disis ML. Variation in the incidence and magnitude of tumor-infiltrating lymphocytes in breast cancer subtypes: a systematic review. JAMA Oncol. 2016; 10.1001/jamaoncol.2016.1061 27355489

[51] Schmidt M, Bohm D, von Torne C, Steiner E, Puhl A, Pilch H (2008). The humoral immune system has a key prognostic impact in node-negative breast cancer. Cancer Res.

[52] Pigott E, Mandik-Nayak L (2012). Addition of an indoleamine 2,3,-dioxygenase inhibitor to B cell-depletion therapy blocks autoreactive B cell activation and recurrence of arthritis in K/BxN mice. Arthritis Rheum.

[53] Adikari SB, Lian H, Link H, Huang YM, Xiao BG (2004). Interferon-gamma-modified dendritic cells suppress B cell function and ameliorate the development of experimental autoimmune myasthenia gravis. Clin Exp Immunol.

[54] Dieci MV, Griguolo G, Miglietta F, Guarneri V (2016). The immune system and hormone-receptor positive breast cancer: is it really a dead end?. Cancer Treat Rev.

[55] Soliman HH, Jackson E, Neuger T, Dees EC, Harvey RD, Han H (2014). A first in man phase I trial of the oral immunomodulator, indoximod, combined with docetaxel in patients with metastatic solid tumors. Oncotarget.

[56] Soliman HH, Minton SE, Han HS, Ismail-Khan R, Neuger A, Khambati F (2016). A phase I study of indoximod in patients with advanced malignancies. Oncotarget.

[57] TC Gangadhar, Hamid O, DC Smith, TM Bauer, JS Wasser, AJ Olszanski, JJ Luke, AS Balmanoukian, DR Kaufman, Y. Zhao, J. Maleski, M.J. Jones, L. Leopold, T.F. Gajewski. Epacadostat plus pembrolizumab in patients with advanced melanoma and select solid tumors: Updated phase 1 results from ECHO-202/KEYNOTE-037. European Society for Medical Oncology Congress (ESMO). Copenhagen, Denmark; 2016.

[58] Zakharia Y MR, Shaheen M, et al. Interim analysis of the Phase 2 clinical trial of the IDO pathway inhibitor indoximod in combination with pembrolizumab for patients with advanced melanoma. American Association for Cancer Research (AACR) Annual Meeting. Washington, DC; 2017.

[59] Primo Lara TMB, O Hamid, David C. Smith, Thomas Gajewski, Tara C. Gangadhar, Bradley G. Somer, Emmett V. Schmidt, Yufan Zhao, Hema Gowda, Anthony J. Olszanski. Epacadostat plus pembrolizumab in patients with advanced RCC: Preliminary phase I/II results from ECHO-202/KEYNOTE-037. American Society of Clinical Oncology (ASCO) Annual Meeting. Chicago, IL; 2017.

[60] David C. Smith TG, O Hamid, JS. Wasser, AJ. Olszanski, SP. Patel, R Mamtani, EV. Schmidt, Y Zhao, JE. Maleski, TC. Gangadhar. Epacadostat plus pembrolizumab in patients with advanced urothelial carcinoma: Preliminary phase I/II results of ECHO-202/KEYNOTE-037. American Society of Clinical Oncology (ASCO) Annual Meeting. Chicago, IL; 2017.

[61] Tara C. Gangadhar BJS, TM Bauer, JS Wasser, AI Spira, SP Patel, AS Balmanoukian, J Bauml, EV Schmidt, Y Zhao, MM Jones, Ahmad A. Tarhini. Efficacy and safety of epacadostat plus pembrolizumab treatment of NSCLC: Preliminary phase I/II results of ECHO-202/KEYNOTE-037. American Society of Clinical Oncology (ASCO) Annual Meeting. Chicago, IL; 2017.

