# Supplementary material for: Objective measurement and clinical significance of IDO1 protein in hormone receptor-positive breast cancer
Source: J Immunother Cancer. 2017 Oct 17;5:81. doi: 10.1186/s40425-017-0285-7 (PMC5644103; doi:10.1186/s40425-017-0285-7)
Supplement: Supplementary file 1 [file 40425_2017_285_MOESM1_ESM.docx]

## Slide 1

SUPPLEMENTAL TABLE 1

## Slide 2

SUPPLEMENTAL FIGURE 1

## Slide 3

SUPPLEMENTAL FIGURE 2

## Slide 4

SUPPLEMENTAL FIGURE 3

## Slide 5

SUPPLEMENTAL FIGURE 4

TILs-high

TILs-low

DAPI/CK/CD3/CD8/CD20 

## Slide 6

SUPPLEMENTAL FIGURE 5

## Slide 7

SUPPLEMENTAL FIGURE 6 

A

DAPI / CK / FOXP3
